# Heterogenous nuclear ribonucleoprotein Q increases protein expression from HIV-1 Rev-dependent transcripts

**DOI:** 10.1186/1743-422X-10-151

**Published:** 2013-05-16

**Authors:** Michelle Vincendeau, Daniel Nagel, Jara K Brenke, Ruth Brack-Werner, Kamyar Hadian

**Affiliations:** 1 Institute of Virology, Helmholtz Zentrum München, Research Center for Environmental Health, Ingolstaedter Landstraße 1, 85764, Neuherberg, Germany; 2 Institute of Molecular Toxicology and Pharmacology/Assay Development and Screening Platform - Helmholtz Zentrum München, Research Center for Environmental Health, Ingolstaedter Landstraße 1, 85764, Neuherberg, Germany

**Keywords:** Rev, hnRNP Q, SYNCRIP, HIV-1 replication, Posttranscriptional regulation

## Abstract

**Background:**

Heterogenous nuclear ribonucleoproteins (hnRNPs) control many processes of the gene expression machinery including mRNA transcription, splicing, export, stability and translation. Recent data show interaction of the HIV-1 Rev regulatory protein with a subset of hnRNP proteins, that includes hnRNP Q, suggesting that hnRNPs can contribute to regulation of HIV-1 gene expression by Rev.

**Findings:**

In this work we address the effect of hnRNP Q on Rev-dependent gene expression. We show that hnRNP Q overexpression increased levels of proteins produced from a Rev-dependent reporter gene in the presence of Rev. Increased protein levels did not correlate with changes in either the levels or the nucleocytoplasmic distribution of Rev-dependent reporter mRNAs. Similar observations were made in persistently HIV-1 infected HeLa cells. In these cells, hnRNP Q overexpression increased levels of the HIV-1 Gag-p24 protein, while levels of viral Rev-dependent mRNAs were not affected.

**Conclusion:**

Our data indicate that hnRNP Q can stimulate the protein production of Rev-dependent mRNAs without changing mRNA levels and mRNA export, respectively. This suggests that hnRNP Q can boost HIV gene expression at the level of protein production.

## Findings

HIV-1 Rev is critical for HIV-1 replication. Rev recognizes the Rev response element (RRE) in partly and unspliced viral RNAs. Binding of Rev to the RRE counteracts negative effects of instability elements (INS) in viral RNA molecules and promotes their transport to the cytoplasm [1-3]. Additionally, the Rev protein is involved in many other processes, e.g. RNA splicing [4], translation of viral RNAs [5,6] and packaging of viral particles [7]. Rev comprises different functional domains responsible for RNA binding/nuclear localization (nuclear localization signal, NLS) [8], nuclear export/transactivation (nuclear export signal, NES) [9] and multimerization of the Rev-protein (oligomerization signals) [2]. Rev interacts with many cellular partners, including Exportin 1 (CRM1) and host cell RNA binding factors like DEAD/H box proteins [10]. In addition, we recently showed that the N-terminus of Rev is able to interact with several hnRNPs [11], a set of RNA binding proteins involved in multiple cellular processes [12]. These interactions seem to play an important role in connecting the diverse functions of Rev during viral gene expression with cellular processes.

The human hnRNP Q protein (synonym: SYNCRIP, Gry-rbp, NSAP1) exists in three isoforms (Q1, Q2 and Q3), which are generated from the same gene by alternative splicing [13]. The full-length protein (Q3) consists of 623 amino acids and contains three RNA-binding domains (RBD), two NLS and a C-terminal Arginine-rich motif (RGG-domain) [13]. HnRNP Q is important for efficient pre-mRNA splicing [13] and can impact mRNA stability [14,15]. Furthermore, hnRNP Q is capable of influencing IRES-dependent mRNA translation [16-18]. In the HIV system, hnRNP Q was recently shown to be part of a group of cellular proteins that binds to a segment of HIV-1 RNA containing splice acceptor site A7 [19] located near the RRE. Interestingly, the group of proteins binding to this region also included translational factors. In a recent study we could demonstrate that hnRNP Q interacts with HIV-1 Rev and is able to positively affect HIV replication, with knockdown of hnRNP Q decreasing viral production [11]. In this Short Report we now present additional research data that strengthen the previously published positive effects of hnRNP Q on HIV-1 replication.

**Figure 1:**
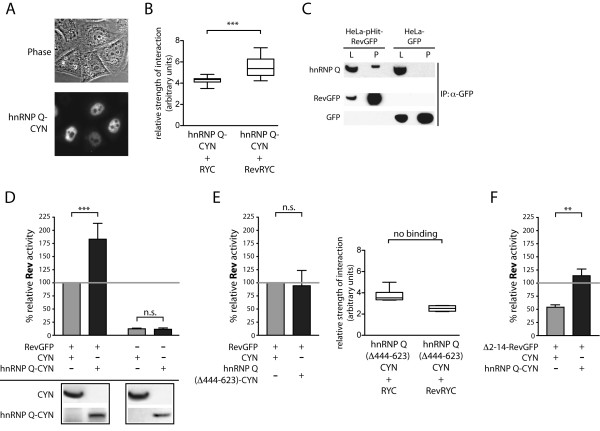
**HnRNP Q induces Rev-dependent reporter protein production. A**) Expression of hnRNP Q-CYN in HeLa cells. **B**) Rev-mRFP-YFP-C (Rev-RYC) and hnRNP Q-CFP-YFP-N (hnRNP Q-CYN) significantly interact in exBIFC assays in HeLa cells. **C**) Immunoprecipitation of Rev-GFP with an anti-GFP antibody specifically co-precipitates endogenous hnRNP Q. **D**) HeLaTatROD cells stably expressing Tat and a Rev-dependent reporter construct were used to evaluate the effect of hnRNP Q on Rev-dependent protein expression. Co-expression of Rev and hnRNP Q-CYN increased reporter protein production by approx. 2-fold compared to the control (CYN). Importantly, this effect was not detectable in the absence of Rev. Expression of hnRNP Q-CYN and CYN proteins were detected by Western Blotting using an anti-GFP antibody. **E**) An hnRNP Q deletion mutant lacking aa 444–623 (hnRNP Q (Δ444-623)-CYN) was not able to enhance Rev-dependent reporter protein production (left graph). Moreover, this mutant lacks the capacity to bind Rev in exBIFC assays (right graph). (**F**) A Rev mutant lacking the amino acids 2–14 (Δ2-14RevGFP) showed a 50% decreased activity when compared to Rev-GFP. This activity was enhanced by ectopic expression of hnRNP Q. Bars represent the means of at least 4 independent experiments and standard deviations are indicated. p-values: *** = p ≤ 0.001; n.s. = not significant (p > 0.05).

We first used a Rev-dependent reporter assay [3,20] to investigate the influence of hnRNP Q on Rev-dependent reporter protein production. Assays were performed in HeLaTatROD cells that contain a stably integrated Rev-reporter gene encoding the *Ds*Red protein and constitutively produce Tat for LTR-dependent transcription of the reporter gene [3]. HeLaTatROD cells were co-transfected with expression plasmids containing the full-length hnRNP Q cDNA sequence fused to sequences encoding a cyan fluorescent tag (pC-hnRNP Q-CYN) and with plasmids for expression of HIV-1 RevGFP (pCsRevsg143; [21]). Expression was verified in HeLa cells (Figure 1A), with hnRNP Q-CYN showing the steady-state nuclear localization pattern described for hnRNP Q [13]. Control transfections were performed in parallel with plasmids (pC-CYN; [11]) expressing only the cyan fluorescent tag. Transfections were performed with the FugeneHD transfection reagent (Roche). Interaction of hnRNP Q with Rev was demonstrated by two assays, i.e. exBIFC (extended bimolecular fluorescence complementation) [22] and co-immunoprecipitation (co-IP) (Figure 1B and C). For exBIFC, Rev was expressed as a fusion protein with the N-terminal half of YFP, while hnRNP Q was linked to the C-terminal half of YFP. The interaction of Rev and hnRNP Q results in complementation of a fluorescing YFP molecule. Co-IP studies were performed with an antibody directed against GFP, thereby precipitating RevGFP containing complexes.

As expected, expression of Rev substantially increased reporter protein production (Figure 1D). Co-expression of RevGFP with hnRNP Q-CYN significantly boosted reporter protein levels even further (approx. 2-fold) compared to the CYN-control (Figure 1D). Similar results were obtained with a plasmid expressing hnRNP Q without the CYN-tag (data not shown), confirming the specific influence of hnRNP Q on reporter protein production. In comparison, hnRNP A1 was not as potent as hnRNP Q to induce reporter protein production (Additional file 1A). Interestingly, hnRNP Q did not increase reporter protein production in the absence of Rev (Figure 1D). In addition, a hnRNP Q deletion mutant lacking the RGG domain from amino acids 444–623 (hnRNP Q-(Δ444-623)-CYN) was not able to boost reporter expression and did not bind Rev in exBIFC assays (Figure 1E). As a control, we explored the effect of hnRNP Q on a GagCTE reporter [23]. This reporter drives Gag production under the control of a LTR promoter and its expression is independent of Rev, but rather uses the TAP/CTE pathway. We could detect a small increase in Gag production through the GagCTE reporter, but notably less pronounced when compared to our Rev-dependent reporter construct (compare Figure 1D and Additional file 2). Further, a Rev mutant lacking the N-terminal 14 amino acids (Δ2-14-Rev), a region involved in the binding to different hnRNPs [11], showed reduced ability to induce Rev-dependent reporter protein production. Surprisingly, hnRNP Q was able to further increase levels of reporter proteins driven by the Δ2-14-Rev mutant indicating that hnRNP Q may also affect reporter protein production by mechanisms independent of Rev-hnRNP Q interaction (Figure 1F). Taken together, our data demonstrate that hnRNP Q is capable of enhancing Rev-mediated reporter protein production.

**Figure 2:**
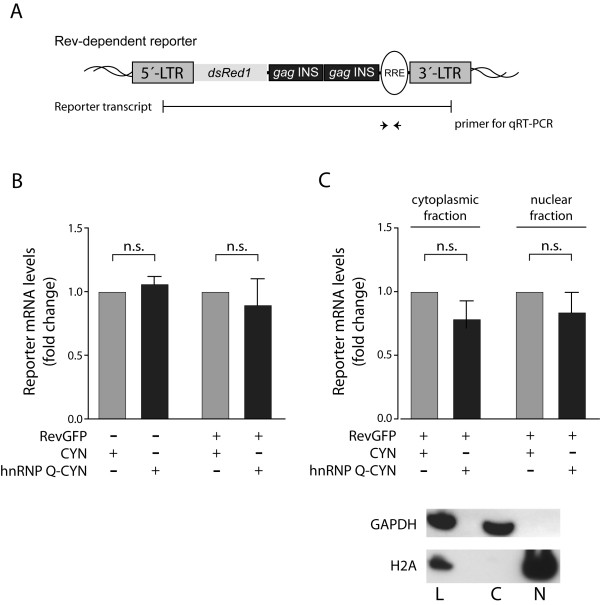
**Overexpression of hnRNP Q does not affect the levels and the nucleocytoplasmic distribution of Rev-dependent reporter mRNAs. A**) Schematic organization of the Rev-dependent reporter construct. The 5′-LTR directs transcription of the *Ds*Red reporter gene. The reporter mRNA contains Rev-dependent regulatory elements (i.e. *gag*-derived instability elements and a Rev-response element (RRE)). Primers for qRT-PCR analyses bind within the RRE region. **B**) Total reporter RNA levels were investigated after expression of hnRNP Q-CYN or the CYN control in the presence or absence of Rev. Overexpression of hnRNP Q did not change total levels of reporter mRNA independent of Rev expression. **C**) Reporter RNA levels were also analyzed after separation of cellular lysates into cytoplasmic and nuclear fractions. Overexpression of hnRNP Q did not enhance export of reporter mRNAs. H2A and GAPDH were used to evaluate purity of cytoplasmic and nuclear fractions, respectively. Bars represent the means of at least 3 independent experiments and standard deviations are indicated. p-values: n.s. = not significant (p > 0.05).

To further analyze reporter activation by hnRNP Q, we evaluated the effects of hnRNP Q on mRNA levels. Again HeLaTatROD cells were co-transfected with a RevGFP expression plasmid together with CYN and hnRNP Q-CYN expression plasmids, respectively. Total RNAs were extracted, reverse transcribed into cDNA (Invitrogen Superscript cDNA reverse transcription kit) and analyzed by quantitative real-time PCR as previously described [21] using specific primers for amplification of the reporter RNAs (forward primer 5′-CGAGCTCGGTACCCCAAGGCAAAGAGAAGAGTGG-3′; reverse primer 5′-CAATAGCCCTCAGCAAATTGTTCTGCTGC-3′) (Figure 2A). For data analysis, the Cp values were normalized to the levels of human RNA Polymerase II. Our results show that reporter mRNA levels are not significantly affected by hnRNP Q expression when compared to the CYN control. This observation was independent of the expression of Rev (Figure 2B). Next, we analyzed the impact of hnRNP Q on nuclear export of reporter mRNAs. To this end, extracts of transfected cells (expressing RevGFP together with hnRNP Q-CYN or with the CYN control) were separated into nuclear and cytoplasmic fractions using the PARIS Kit (Ambion). RNAs from both fractions were individually purified, reverse transcribed and reporter transcripts were analyzed by quantitative RT-PCR as described above. Purity of nuclear and cytoplasmic fractions was verified by Western Blot analysis using histone H2A as a nuclear and GAPDH as a cytoplasmic marker. Both fractions showed fairly no cross-contamination (Figure 2C). Investigation of nuclear and cytoplasmic reporter mRNAs by qRT-PCR revealed no increase of reporter transcripts in the cytoplasmic compartment after hnRNP Q overexpression compared to the CYN control (Figure 2C), suggesting that hnRNPQ overexpression does not influence nuclear export of reporter mRNAs. In contrast, hnRNP A1 clearly increased levels of nuclear reporter mRNA (Additional file 1B).

**Figure 3:**
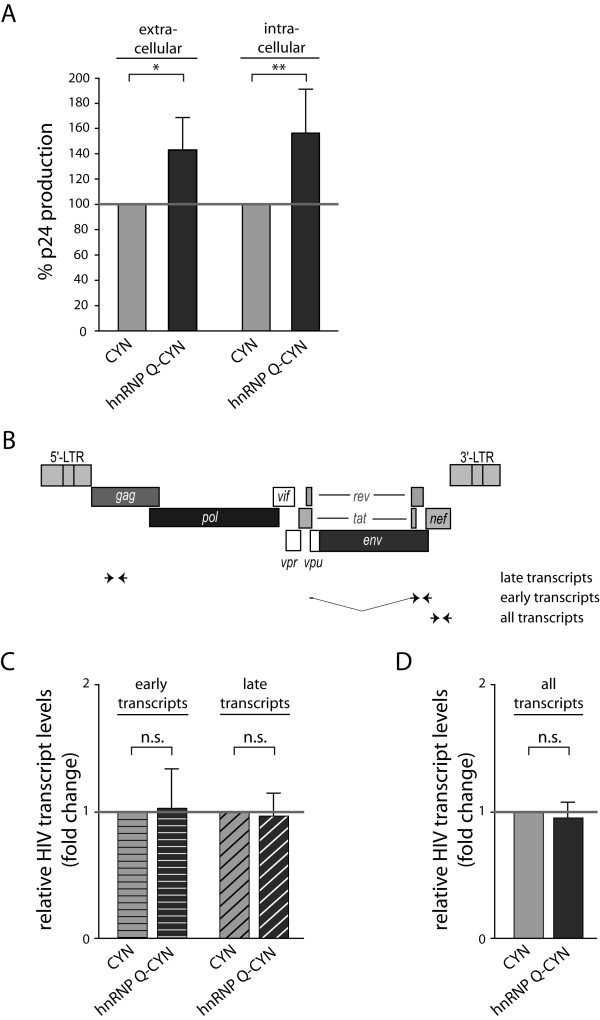
**HnRNP Q overexpression elevates Gag-p24 production without affecting levels of HIV-1 transcripts. A**) Chronically HIV-1 infected HeLa cells (LC5-HIV) were used to evaluate the effects of hnRNP Q overexpression on HIV-1 replication. Using ELISA, p24 protein levels were analyzed in LC5-HIV cells ectopically expressing hnRNP Q-CYN or CYN. The results revealed that both extra- and intracellular p24 protein levels are elevated by hnRNP Q overexpression. **B**) Specific primers (indicated in the scheme of the HIV-1 genome) were designed to distinguish between Rev-dependent late transcripts (i.e. unspliced and singly-spliced), and Rev-independent early transcripts (i.e. completely spliced) and all transcripts. The primers for detection of late transcripts were placed into the *gag* ORF and for detection of all transcripts into the *nef* ORF. We used a 5′ spanning primer bridging the two *rev* exons and a 3′ primer located at the end of the second *rev* exon to quantify early transcripts by qRT-PCR. **C**) Quantitative analyses of HIV transcripts showed that levels of neither early nor late transcripts are affected by ectopic expression of hnRNP Q in chronically HIV-1 infected cells. **D**) Analysis of total levels of HIV-1 transcripts confirmed that overexpression of hnRNP Q did not affect HIV-1 transcript levels. p-values: * = p ≤ 0.05; ** = p ≤ 0.01; n.s. = not significant (p > 0.05).

To study the effects of hnRNP Q in the context of complete HIV-1 replication, we analyzed the influence of hnRNP Q overexpression on levels of p24Gag produced by persistently HIV-1 infected HeLa cells (LC5-HIV) [24]. p24 protein levels were quantified by ELISA as previously described [11,21]. In line with our data showing increased Rev-dependent reporter protein production by hnRNP Q (Figure 1D), ectopic hnRNP Q expression significantly increased intra- and extracellular p24 protein levels by LC5-HIV cells (Figure 3A). Hence, hnRNP Q is also able to enhance production of late viral gene products in a persistently infected cell system. A slight increase in p24 protein production was also detected after ectopic expression of hnRNP A1 (Additional file 1C). To evaluate whether hnRNP Q alters the levels of Rev-dependent HIV-1 transcript classes, we quantified levels of different HIV-1 transcript species in LC5-HIV cells expressing hnRNP Q-CYN. To this end we performed qRT-PCR with primers (Figure 3B) that specifically amplify either Rev-dependent late transcripts (i.e. unspliced and singly spliced RNAs: forward primer 5′-GCCCCTCCCATCAGTGGAC-3′ and reverse primer 5′- GCCTTGGTGGGTCGTACTCCTAATGG-3′) or Rev-independent early transcripts (i.e. completely spliced RNAs: forward primer 5′-CTCTATCAAAGCAACCCACCTCCCAA-3′ and reverse primer 5′-GCGGTGGTAGCTGAAGAGGCACAGG-3′) or all HIV-1 transcripts (forward primer 5′-CCAGTCACACCTCAGGTACCTTTAAGACC-3′ and reverse primer 5′-GTGTGTGGTAGATCCACAGATCAAGG-3′). HIV-1 transcript levels were normalized to human RNA Polymerase II mRNA levels. The results of these qRT-PCR analyses demonstrate that hnRNP Q does not significantly alter levels of Rev-dependent or Rev-independent transcript classes (Figure 3C) or of total HIV-1 transcripts (Figure 3D). These results indicate that hnRNP Q overexpression does not affect quantities of Rev-dependent mRNAs in HIV-producing cells, and agree with the results of the analysis of reporter transcripts (Figure 2B).

In this Short Report we addressed the effect of hnRNP Q on Rev-dependent reporter production and HIV-1 replication. For overexpression we used the full-length hnRNP Q coding sequence. Overexpression of hnRNP Q increased protein levels produced from Rev-dependent transcripts without affecting amounts or nucleocytoplasmic distribution of Rev-dependent transcripts. This point is remarkable as hnRNP Q is known to influence Hepatitis C virus RNA replication [25], splicing events [13] and mRNA stability [14,15]. The increase in p24 production is congruent with our recent findings that knockdown of hnRNP Q in persistently infected astrocytes leads to diminished p24 production [11]. Of note, the influences by hnRNP Q on the Rev-dependent reporter protein production were visibly higher when compared to a GagCTE reporter indicating a specific effect of hnRNP Q on the Rev/RRE axis. Moreover, hnRNP Q had a bigger effect on Rev-dependent reporter protein production than hnRNP A1 and the effects by these two hnRNPs were distinct of each other. While hnRNP Q did not affect mRNA levels, hnRNP A1 markedly increased reporter mRNA levels in the nucleus. One possibility to explain these hnRNP A1 effects is that hnRNP A1 is able to stabilize INS containing mRNAs [26]. Over time the elevated number of reporter mRNAs lead to an increased protein production. However, hnRNP A1 has also other influences on viral replication (e.g. on the level of splicing) that could lead to the induced reporter protein and p24 production.

Interestingly, the influence of hnRNP Q on protein production from a Rev dependent reporter gene was only visible in the presence of HIV-1 Rev. Rev interaction with hnRNP Q was shown to involve the N-terminus of Rev [11]. Surprisingly, we still see a positive effect of hnRNP Q on the activity of a Rev-deletion mutant lacking the N-terminus. This could be due to secondary binding sites in Rev for hnRNP Q since we previously reported that deletion of the N-terminus of Rev significantly diminished, but did not abrogate binding of Rev to hnRNP Q [11]. However, we cannot exclude that there is also an activity of hnRNP Q on HIV-1 protein production independent of direct binding to Rev. In future studies we will decipher the mechanisms behind the mutual as well as individual impacts of Rev and hnRNP proteins to regulate HIV-1 replication.

Recently the Rev co-factors Sam68, eIF5A, hRIP and DDX3, which are essential for nuclear export of Rev-dependent mRNAs [7], were shown to be able to regulate HIV-1 replication on the translational level [27]. This expands the impact of multifunctional Rev-interaction partners to processes in the cytoplasm. Our new data suggest that hnRNP Q could also be involved in the translational control of HIV-1 replication. Indeed, hnRNP Q was shown to influence translation of different mRNAs, including HCV mRNA [17], BiP mRNA [16] and Rev-erb alpha within circadian oscillation [18] proving its presence and function in the cytoplasmic compartment. The very diverse set of hnRNP proteins comprise a variety of cellular functions (mRNA transcription, splicing, trafficking, stability, translation, etc.) inside the nucleus as well as the cytoplasm [28,29] and many hnRNPs are known to regulate HIV-1 replication (discussed in [11] and [30,31]). Thus, hnRNP Q may also recruit other hnRNPs to co-activate HIV-1 replication. Future studies will address the role of hnRNP Q in the translation of HIV-1 proteins.

Taken together, we demonstrate supporting data that hnRNP Q increases p24 production in persistently infected cells and Rev-dependent reporter protein production. These effects are not associated with changes in RNA levels or nucleocytoplasmic distribution of Rev-dependent mRNAs, suggesting that hnRNP Q can contribute to positive effects on HIV-1 protein production.

## Competing interests

The authors declare that they have no competing interests.

## Authors’ contributions

MV, DN, RBW and KH designed the experiments. MV, DN, JB and KH performed the experiments. RBW and KH wrote the manuscript. All authors read and approved the final manuscript.

## Supplementary Material

Additional file 1 **HnRNP A1 induces protein production of Rev-dependent mRNAs and stabilizes reporter mRNAs in the nucleus.** (A) HeLaTatROD cells were co-transfected with plasmids for expression of Rev and hnRNP A1-CYN or CYN. An increase in reporter protein production of approx. 25-30% by hnRNP A1-CYN was observed compared to the control (CYN). (B) Reporter RNA levels were analyzed in cytoplasmic and nuclear fractions. Ectopic expression of hnRNP A1 notably elevated levels of reporter mRNAs in the nuclear fraction. (C) In chronically HIV-1 infected HeLa cells, hnRNP A1 overexpression was able to slightly increase p24 protein levels in extra- and intracellular fractions. Click here for file

Additional file 2 **HnRNP Q overexpression has little effects on a GagCTE reporter.** HeLa cells were co-transfected with plasmids for GagCTE reporter and Tat expression together with hnRNP Q-CYN or the control CYN. The relative GagCTE reporter activity was slightly induced after ectopic hnRNP Q expression. Click here for file
