# Supplementary figures and images for: Heterogenous nuclear ribonucleoprotein Q increases protein expression from HIV-1 Rev-dependent transcripts
Source: Virol J. 2013 May 16;10:151. doi: 10.1186/1743-422X-10-151 (PMC3673855; doi:10.1186/1743-422X-10-151)

# Additional File 1

A

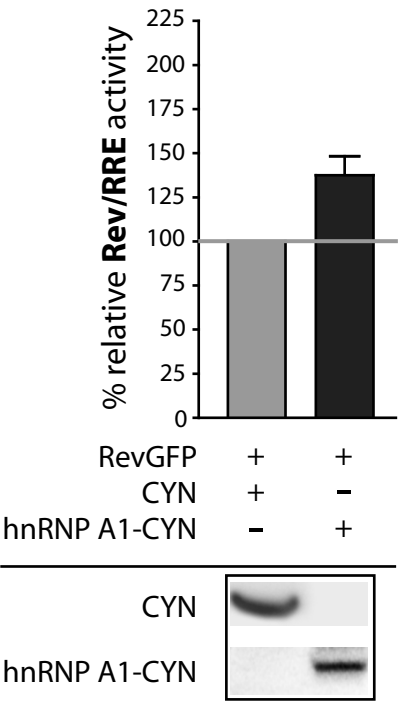

B

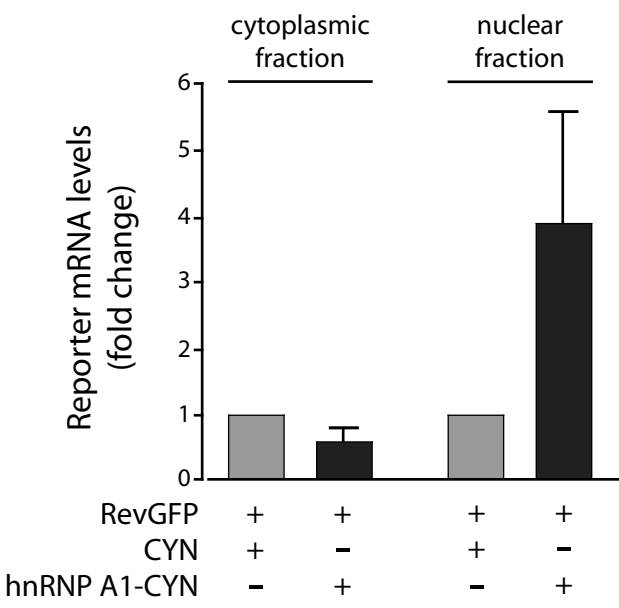

C

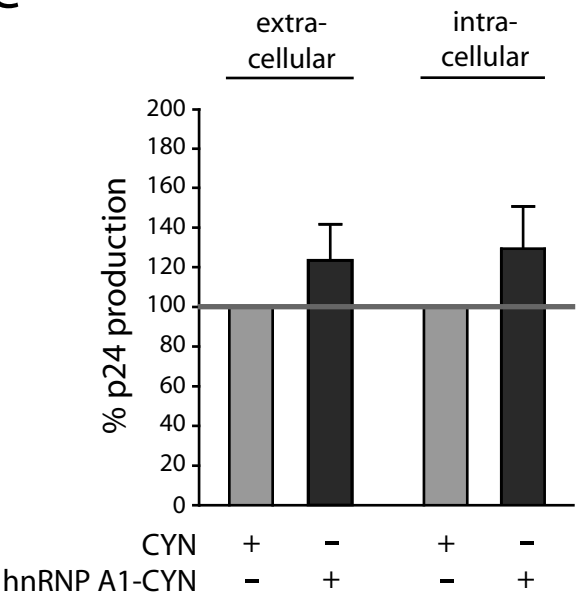

Supplement: Additional file 1 — HnRNP A1 induces protein production of Rev-dependent mRNAs and stabilizes reporter mRNAs in the nucleus. (A) HeLaTatROD cells were co-transfected with plasmids for expression of Rev and hnRNP A1-CYN or CYN. An increase in reporter protein production of approx. 25-30% by hnRNP A1-CYN was observed compared to the control (CYN). (B) Reporter RNA levels were analyzed in cytoplasmic and nuclear fractions. Ectopic expression of hnRNP A1 notably elevated levels of reporter mRNAs in the nuclear fraction. (C) In chronically HIV-1 infected HeLa cells, hnRNP A1 overexpression was able to slightly increase p24 protein levels in extra- and intracellular fractions. [file 1743-422X-10-151-S1.pdf]

## Additional File 2

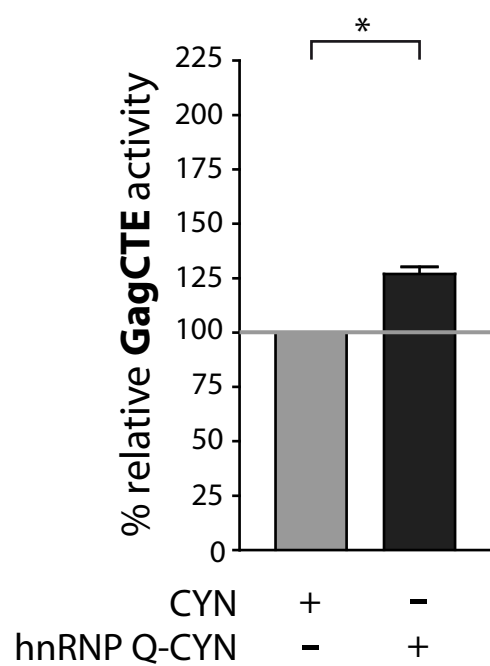

Supplement: Additional file 2 — HnRNP Q overexpression has little effects on a GagCTE reporter. HeLa cells were co-transfected with plasmids for GagCTE reporter and Tat expression together with hnRNP Q-CYN or the control CYN. The relative GagCTE reporter activity was slightly induced after ectopic hnRNP Q expression. [file 1743-422X-10-151-S2.pdf]
